# Juvenile myoclonic epilepsy has hyper dynamic functional connectivity in the dorsolateral frontal cortex

**DOI:** 10.1016/j.nicl.2018.11.014

**Published:** 2018-11-19

**Authors:** Yanlu Wang, Ivanka Savic Berglund, Martin Uppman, Tie-Qiang Li

**Affiliations:** a Department of Clinical Science, Intervention, and Technology, Karolinska Institute, Stockholm, Sweden; b Department of Medical Radiation Physics and Nuclear Medicine, Karolinska University Hospital, Sweden; c Department of Women's and Children's Health, Karolinska Institute, Stockholm, Sweden; d Department of Neurology, Karolinska University Hospital, Sweden; e Department of Neurology, David Geffen School of Medicine, University of California, Los Angeles, USA

**Keywords:** Epilepsy, Juvenile myoclonic epilepsy, Resting-state fMRI, Neuroimaging, Temporal analysis

## Abstract

**Purpose:**

Characterize the static and dynamic functional connectivity for subjects with juvenile myoclonic epilepsy (JME) using a quantitative data-driven analysis approach.

**Methods:**

Whole-brain resting-state functional MRI data were acquired on a 3 T whole-body clinical MRI scanner from 18 subjects clinically diagnosed with JME and 25 healthy control subjects. 2-min sliding-window approach was incorporated in the quantitative data-driven data analysis framework to assess both the dynamic and static functional connectivity in the resting brains. Two-sample *t*-tests were performed voxel-wise to detect the differences in functional connectivity metrics based on connectivity strength and density.

**Results:**

The static functional connectivity metrics based on quantitative data-driven analysis of the entire 10-min acquisition window of resting-state functional MRI data revealed significantly enhanced functional connectivity in JME patients in bilateral dorsolateral prefrontal cortex, dorsal striatum, precentral and middle temporal gyri. The dynamic functional connectivity metrics derived by incorporating a 2-min sliding window into quantitative data-driven analysis demonstrated significant hyper dynamic functional connectivity in the dorsolateral prefrontal cortex, middle temporal gyrus and dorsal striatum. Connectivity strength metrics (both static and dynamic) can detect more extensive functional connectivity abnormalities in the resting-state functional networks (RFNs) and depict also larger overlap between static and dynamic functional connectivity results.

**Conclusion:**

Incorporating a 2-min sliding window into quantitative data-driven analysis of resting-state functional MRI data can reveal additional information on the temporally fluctuating RFNs of the human brain, which indicate that RFNs involving dorsolateral prefrontal cortex have temporal varying hyper dynamic characteristics in JME patients. Assessing dynamic along with static functional connectivity may provide further insights into the abnormal function connectivity underlying the pathological brain functioning in JME.

## Highlights

• JME patients had hyper-connectivity in dorsolateral prefrontal cortex, dorsal striatum, and middle temporal gyri.
• Sliding window analysis captured more brain regions with enhanced functional connectivity.
• The dynamic functional connectivity produces both larger and more distinct brain regions of hyper-connectivity.
• Dynamic analysis also produces more statistically significant group analysis results.

## 1 Introduction

In the last two decades resting-state functional magnetic resonance imaging (resting-state functional MRI) has emerged as a useful method to identify the neuropathological signatures for different subtypes of epilepsy (Centeno and Carmichael, 2014; Chen et al., 2017; Gupta et al., 2017; Paldino et al., 2017; Peng and Hsin, 2017; Wang et al., 2017). For example, alterations in the default mode network (DMN) and thalamus were detected in childhood absence epilepsy (Wang et al., 2017); The thalamus was shown to be impacted in patients suffering from generalized temporal lobe epilepsy (Peng and Hsin, 2017), while the basal ganglia was strongly implicated in frontal lobe epilepsy (Dong et al., 2016a, Dong et al., 2016b). For patients with juvenile myoclonic epilepsy (JME) functional connectivity disturbances in both the thalamo-motor and thalamo-frontal networks were found (Dong et al., 2016b; Jiang et al., 2018). Resting-state functional MRI may even be useful for identifying subjects at increased risk to develop epilepsy (Gupta et al., 2017). Most of the previous Resting-state functional MRI studies of epilepsy patients have assessed the static functional connectivity, which refers to the functionally integrated relationship between spatially separated brain regions in the entire time period of Resting-state functional MRI data sampling session (Friston, 2011). Static functional connectivity is usually derived from the entire Resting-state functional MRI time courses in forms of temporal correlation of the signal intensities between different brain regions. However, more deliberate studies have recently demonstrated that the resting-state functional networks (RFNs) of the brain fluctuate on the scale of seconds to minutes (Hutchison et al., 2013a, Hutchison et al., 2013b). The concept of dynamic functional connectivity is, therefore, an important aspect of the resting-state functional activities, which considers functional connectivity variations over a short period of time.

Dynamic functional connectivity has now been investigated in a variety of contexts related to both behavior and neural activity (Hutchison et al., 2013a, Hutchison et al., 2013b), which has deepened our understanding of the functional networks in the brain. Numerous studies have shown reproducible patterns of short-term network activity that move throughout the brain (Hutchison et al., 2013a, Hutchison et al., 2013b). Such transition of brain states has been confirmed in both animal models and humans (Majeed et al., 2011). Dynamic functional connectivity analysis has shown that the spontaneous transitions between networks of interacting brain areas are highly organized into a hierarchy of two types of metastates: one for higher order cognition, and the other for the sensorimotor systems (Li et al., 2016). The transitions are more probable within, than between, metastates. Furthermore, the time spent in each metastate is individual specific and associated with behavior (Vidaurre et al., 2017). There is also emerging evidence to indicate that dynamic functional connectivity is affected by a variety of factors, such as mental states (Esposito et al., 2006), sleep (Horovitz et al., 2008), learning (Bassett et al., 2011), and neuropathologies (Chahine et al., 2017; Rashid et al., 2016; Schmidt et al., 2014; Rashid et al., 2014; Bastos-Leite et al., 2015). There has been a rapidly growing interest in utilizing dynamic functional connectivity to study neuropsychiatric disorders where time-varying abnormalities have been reported in different neuropsychiatric diseases, such as depression, schizophrenia, and Alzheimer's diseases. Most of the findings are related to the disruption of some specific function networks and the transition patterns among different metastates. For example, it has been reported that schizophrenia patients have less frequent changes between the implicated RFNs than healthy subjects (Chahine et al., 2017; Rashid et al., 2016; Schmidt et al., 2014; Rashid et al., 2014; Bastos-Leite et al., 2015). A few studies have recently reported hyper dynamic activities in some specific RFNs for different sub-types of epilepsy disorders. For example, abnormal dynamic functional connectivity were detected for temporal lobe epilepsy in hippocampi (Laufs et al., 2014), temporal pole (Nedic et al., 2015), posterior cingulate cortex (Douw et al., 2015), and inter-regional connectivity within DMN (Robinson et al., 2017). Aberrant functional connectivity dynamics in DMN was also observed for idiopathic generalized epilepsy with generalized tonic–clonic seizures (Liu et al., 2017).

As discussed above, the association between the disrupted functional networks and specific neuropathological behaviors for some sub-types of epilepsy disorders has been the focus of many neurophysiological and neuroimaging studies. Such network notion has seminally improved our understanding and characterization of epilepsy. However, to the best of our knowledge, dynamic functional connectivity has not been assessed in JME patients. The focus of the present study is to assess both static and dynamic functional connectivity characteristics of JME subjects in a quantitative data-driven fashion. As the most common idiopathic generalized epilepsy disorder, many issues remain not fully understood in JME. Previous studies have provided multiple lines of evidence to support the hypothesis that JME is a multi-regional thalamocortical ‘network’ epilepsy form (Yacubian, 2017; Loughman et al., 2016), rather than a generalized epilepsy disorder. Furthermore, stationary connectomic correlates with cognitive impairment have been found in the thalamo-motor and thalamo-frontal systems (Dong et al., 2016b; Jiang et al., 2018; Paulus et al., 2015; Vollmar et al., 2011). However, it remains largely unclear whether deficiency is present in the dynamic interactions between RFNs or between brain areas within a given RFN. In this study, we incorporated sliding window analysis of time-varying connectivity hierarchy (SWATCH) into previously developed quantitative data-driven analysis framework (Li et al., 2016; Nordin et al., 2016) to assess functional connectivity both in the entire data acquisition period (assumed stationary) and a 2-min sliding window in a voxel-wise fashion. With the SWATCH analysis, we can derive two types of functional connectivity metrics, namely the functional connectivity strength index (CSI) and connectivity count index (CCI) as well as the temporal means of their sliding-window time series from the acquired resting-state fMRI data. By comparing to the data form the demographically matched healthy controls with voxel-wise *t*-test, we aimed to address the following questions: 1) Is there any JME specific static abnormality in terms of CCI and CSI? 2) Is there any JME specific dynamic functional connectivity abnormality in the terms of the temporal means of CSI and CCI? 3) Is there any spatial overlap between the detected static and dynamic functional connectivity abnormalities? Clarification of these issues may increase the amount of diagnostic information that can be obtained from Resting-state functional MRI measurements in JME. Furthermore, it can improve our understanding of the brain functioning mechanisms in JME regarding to their cognitive impairment.

## 2 Materials and methods

### 2.1 Data acquisition

The Central Ethical Review Board for Stockholm region approved the ethical permission for the Resting-state functional MRI study. For this study, we recruited a total of 25 normal adult subjects (male/female = 14/10, age = 33.2 ± 13.5), and clinically diagnosed JME patients. All recruited patients are long-term consecutive patients at Karolinska University Hospital (Stockholm, Sweden). They were controlled at this hospital from the year of their seizure onset and were well known to the neurologists in charge. Only patients whose seizure phenomenology was reliably assessed with the aid of close relatives were included in the study. All patients were also investigated with high resolution MRI according to the epilepsy protocol, and those with abnormal structural findings were excluded from the study. The diagnosis of JME was based on seizure history, seizure semiology as described by relatives or recorded during hospitalization, and scalp EEG recordings. Eighteen patients (3 males and 15 females, mean age 30.11 ± 7.73 years, range 20–48 years) were diagnosed with JME. They had late childhood-or-teenage onset of awakening myoclonic jerks, often in the upper, but sometimes also in the lower extremities. All of them had history of myoclonies as well as Generalized tonic-clonic seizures. Four of them also experienced rare absences. None of the patients had a progressive condition. The inclusion criterion in education was 11 years in school, of which at least 2 years in non-mandatory education (equivalent to high-school and above). The included patients had on average 12.7 ± 1.7 years in school, and their intelligence was accessed to be not impacted by the disorder to the degree such that mandatory education in Sweden cannot be completed. The demographic, symptomatic, and medical history data are detailed in Supporting Information A.

All MRI scans were conducted using a 3 T whole-body clinical MRI scanner (TIM Trio, Siemens Healthcare, Erlangen, Germany). A single-shot 2D gradient-recalled echo echo-planar imaging (EPI) pulse sequence was used to acquire the Resting-state functional MRI data with the following essential acquisition parameters: 32 transverse slices (3.6 mm thickness), TR/TE = 2000/35 ms, FOV = 220 mm, matrix size = 64 × 64, flip angle = 90°, 300 dynamic time-frames, IPAT = 2. A 32-channel phased-array head coil was used for the signal reception. Foam paddings were used for every subject to reduce the head motions. During the Resting-state functional MRI scans the participants were instructed to close their eyes but not fall asleep, they were also instructed to not think about anything specific during the Resting-state functional MRI scans.

### 2.2 Resting-state functional MRI data preprocessing

All Resting-state functional MRI datasets underwent the same preprocessing procedure, which was performed with AFNI (http://afni.nimh.nih.gov/afni) and FSL (http://www.fmrib.ox.ac.uk/fsl) programs with a bash wrapper shell. The first 5 time-frames in each dataset were removed to ensure signal steady state. After de-spiking, six-parameter rigid body image registration was performed for motion correction. The average volume for each motion-corrected time series was used to generate a brain mask to minimize the inclusion of the extra-cerebral tissue. Spatial normalization to the Montreal Neurological Institute (MNI) standard-space T1-weighted average structural template image was performed using a 12-parameter affine transformation and mutual-information cost function. During the affine transformation, the imaging data were also re-sampled to isotropic resolution using a Gaussian kernel with 4 mm Full Width at Half Maximum (FWHM) as dictated by the largest voxel dimension. Nuisance signal removal was performed by voxel-wise regression using the 6 motion correction parameters, average signal of the ventricles and their respective 1st order derivatives, totaling on 14 regressors. After removing baseline trend up to the third order polynomial, effective band-pass filtering was performed using low-pass filtering at 0.08 Hz. Local Gaussian smoothing up to FWHM = 5 mm was performed using an eroded gray matter mask.

### 2.3 Static functional connectivity with quantitative data-driven analysis

For each subject, the Pearson's cross-correlation coefficients of the resting-state functional MRI time courses were computed for voxel-pairs inside a brain mask, generating a N × N (where N is the number of voxels within the brain mask) correlation coefficient matrix. For data reduction, the following functional connectivity index maps were calculated from the huge correlation coefficient map for each subject: 1) Connection Strength Index (CSI) which is defined as the non-zero mean value of the Pearson correlation coefficient (CC) ≥ 0.3 for all voxel pairs involving the current voxel in question. 2) Connection Count Index (CCI) which is the number of voxel pairs involving the current voxel in question with the absolute CC ≥ 0.3.

### 2.4 Dynamic functional connectivity with SWATCH analysis

**Fig. 1:**
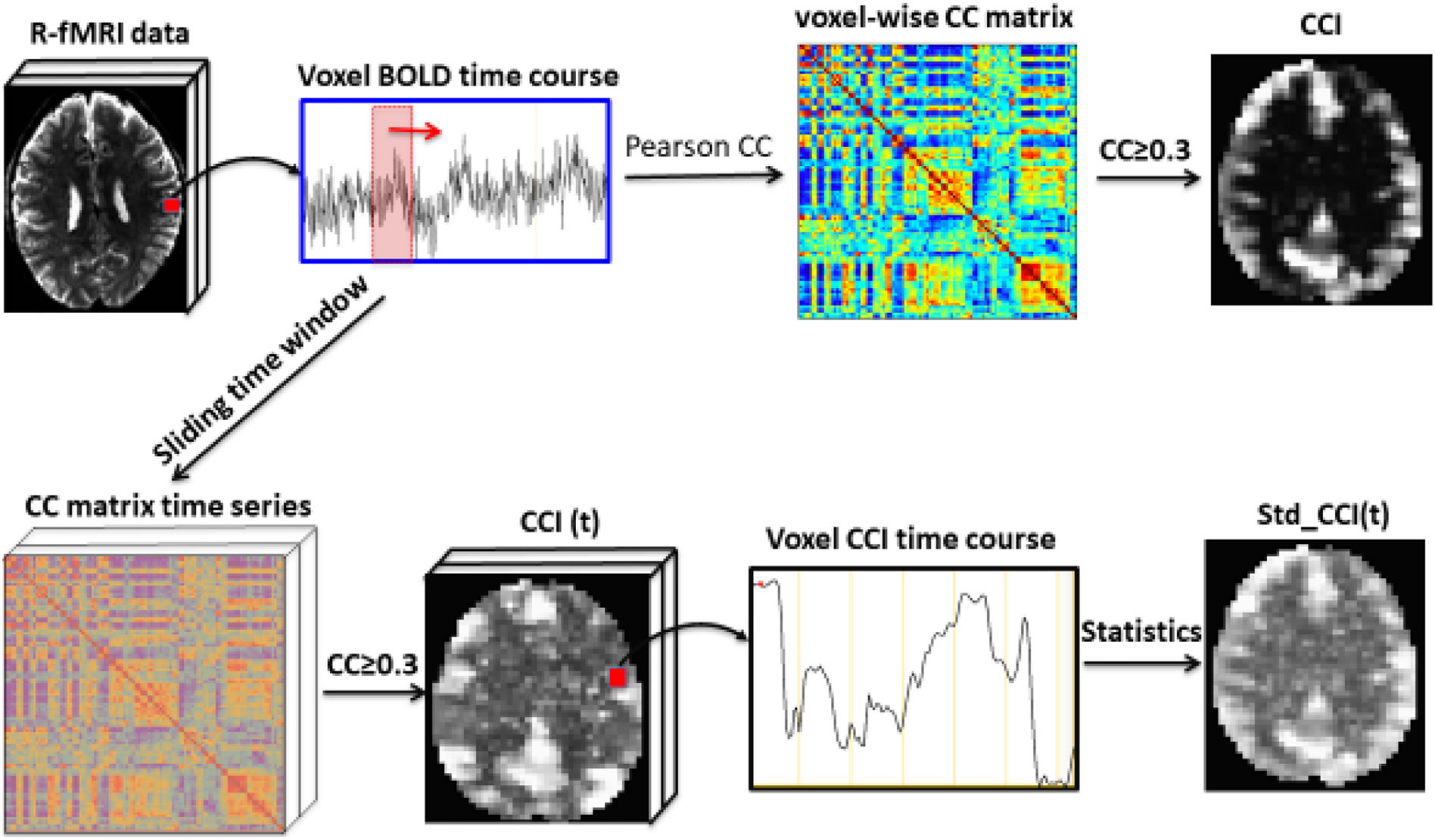
Schematic illustration of the quantitative data analysis (top row) and SWATCH (bottom row) pipelines. With quantitative data-driven analysis the entire time course of the resting-state functional MRI data is used to compute the CC matrix for all voxels within the brain. The CC matrix size is typically in the order of 15 × 103 for Resting-state functional MRI data with 4 mm spatial resolution. For data reduction in quantitative data-driven analysis, we compute the number of correlated voxel-pairs and their average CC values ≥0.3. The threshold was selected based on the empirical assessment of the statistical sensitivity of the derived metrics (Allen et al., 2014). With SWATCH, a 2-min window of the time course was used to compute the CC matrix. While the 2-min window sliding along the time course, a time series of CC matrixes were obtained, from which a time series of metrics can be derived as in quantitative data-driven analysis. The dynamic aspects of the time series of metrics can be evaluated using temporal statistics, such as the temporal means of the time series.

To characterize the time variations of the CCI and CSI maps we incorporated a 2 min sliding window into the quantitative data-driven analysis procedure. We computed CCI and CSI maps for every 2 min window (60 time-frames). The increment between the adjacent sliding windows was one frame, therefore, we obtained a time series of CCI and CSI maps with 235 time-frames for each Resting-state functional MRI dataset. Then, we computed the voxel-wise temporal averages of the 2-min sliding window CCI and CSI time series for each Resting-state functional MRI dataset for further statistical analysis. The pipeline of the SWATCH analysis for 10-min acquisition with a 2-min sliding window is schematically outlined in Fig. 1.

### 2.5 Statistical analysis

Differences in motion during data acquisition between the two groups were accessed by extracting the maximum of each parameter in the transformation matrix across time for each subject and compared between healthy control and JME patients.

To test the possible differences in functional connectivity between JME patients and the healthy controls, we performed un-paired, Welch's 2-way *t*-test of the CCI and CSI data for the entire time course and the temporal averages, <CCI> and < CSI>, corresponding to their respective 2-min sliding window series using the AFNI program *3dttest++*. Age and sex of the individual subjects were included as confounders in the statistical tests. The statistical significance of the *t*-tests was assessed first by setting an uncorrected voxel-wise threshold at *p* < .01 and then imposing a minimum voxel cluster size of at least 20 contiguous voxels. The probability of random field of noise producing a cluster of size ≥ 20 was estimated at family-wise error rate (FWER) *p* < .05. This was concluded from the Monte-Carlo simulation result obtained by using the AFNI program, *AlphaSim+*. The simulations we used following essential input parameters: matrix size = 45 × 45 × 54, a gray matter mask based on MNI template, voxel-wise threshold value *p* < .01, 10^6^ iterations, and FWHM = 5.8 mm, which was the estimated average by applying the AFNI program, 3dFWHMx, to the CSI and CCI image data, which was quite close to FWHM = 5 mm used in the final imaging smoothing procedure described above. On top of the cluster simulation results, any resulting clusters are further filtered with the ETAC (Equitable Thresholding and Clustering) approach, ensuring global false positive rates (FPR) of <5% through (equitable) multiple thresholding of randomization/permutation technique (Cox, 2018). Due to the cluster size filtering was performed prior to ETAC, the resulting clusters may be smaller than 20 voxels in size.

## 3 Results

None of the transformation parameters showed significant differences between JME patients and healthy controls (*p* > .12), indicating that JME patients did not move more than the healthy control subjects during acquisition. The confounders did not result in any regions of significance (*p* < .01) that survived our thresholding scheme (global FPR < 5%), and hence not shown. However, it should be noted that the confounders, especially age, showed regions of significance (p < .01) without cluster thresholding.

**Fig. 2:**
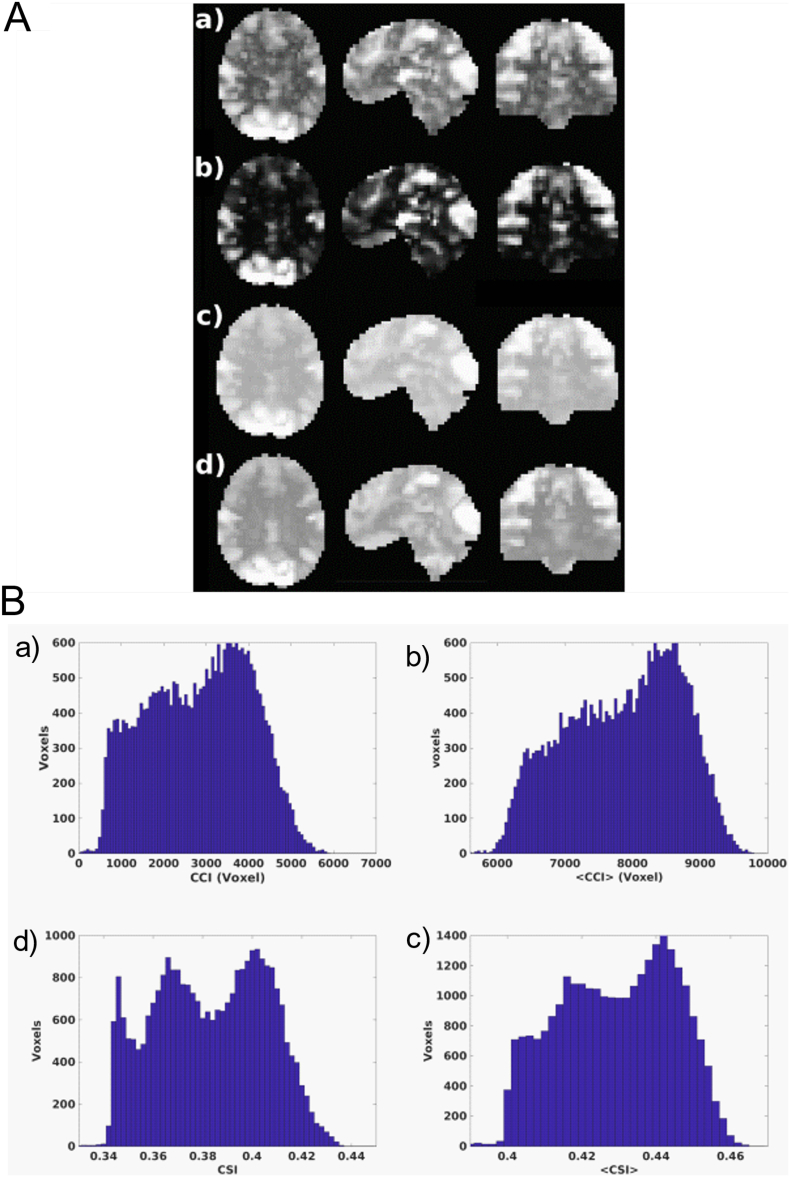
A: Cross-sectional display of the static and dynamic functional connectivity metrics for a JME patient participated in the study. (a) CCI, (b) <CCI>, (c) CSI, (d) <CSI>. These metrics were re-scaled differently to be displayed in the same panel. B: Histograms for the static and dynamic functional connectivity metrics shown in A. (a) static CCI, (b) dynamic <CCI>, (c) static CSI, (d) dynamic <CSI>.

**Fig. 3:**
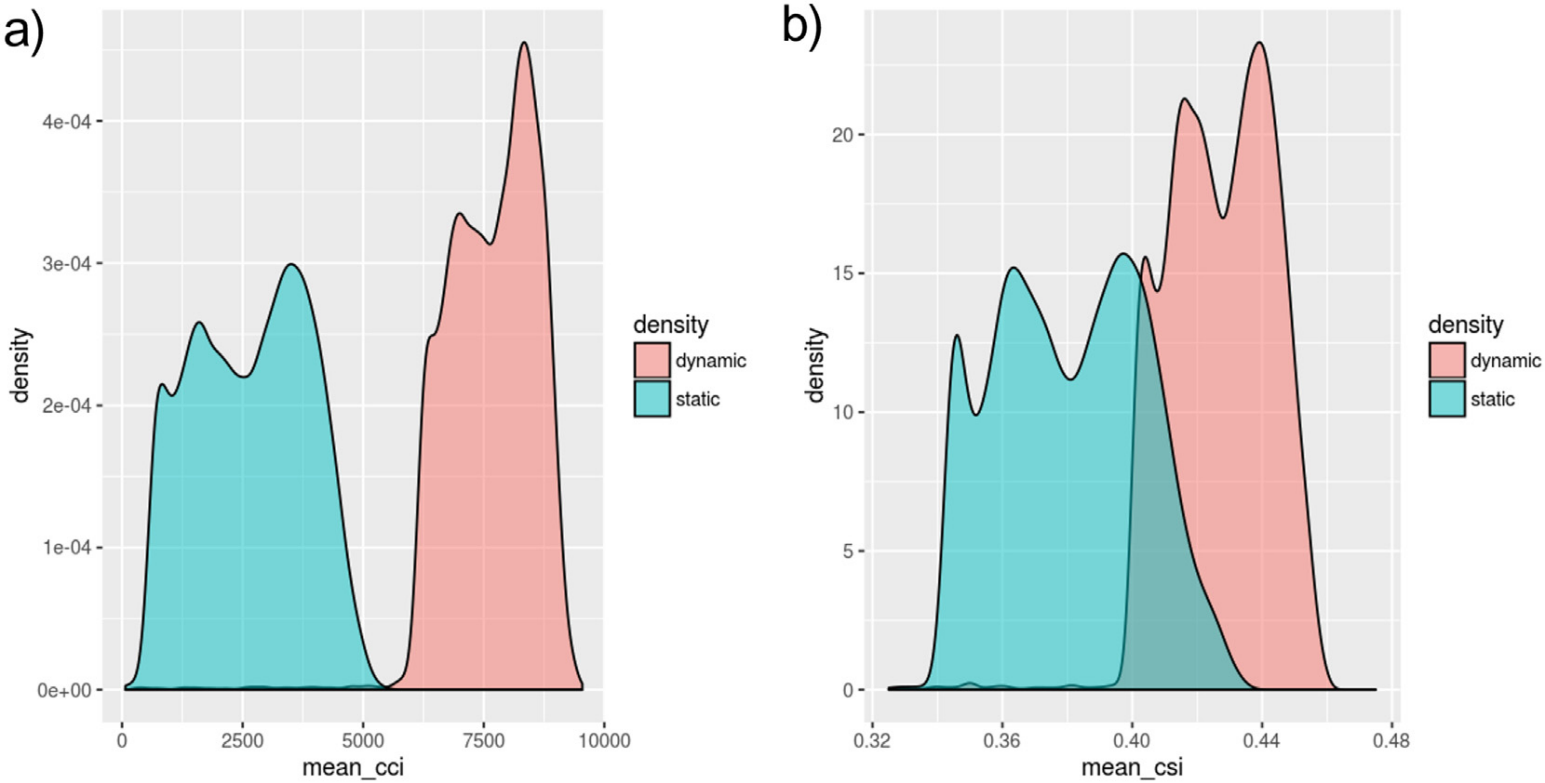
Average density plots for the static and dynamic functional connectivity metrics for all subjects in the study. (a) CCI and <CCI> in turquoise and salmon respectively, (b) CSI and <CSI> in turquoise and salmon respectively. For both metrics, the dynamic metrics possess higher values overall compared to the static metrics. The dynamic metrics also have a higher portion of high values compared to lower values within their own density plot compared to their static counterparts.

Fig. 2A shows a typical set of static and dynamic functional connectivity images for a JME patient participated in the study, as computed from the resting-state functional MRI measurements with the SWATCH analysis method described above. There is an overall consistency in tissue contrast among the different connectivity metrics. The brain regions with high functional connection density and strength both in the entire data acquisition period and 2-min sliding window include the anterior and posterior cingulate, precuneus, insula, visual and sensory motor cortices, which have been previously identified as the major functional connectivity hubs. The contrast differences between the connection density metrics (CCI and <CCI>) and the connection strength metrics (CSI and <CSI>) are also quite noticeable. This can be better appreciated by the corresponding histograms shown in Fig. 2B. The contrast differences can also be seen on density plots of the static and dynamic metrics averaged across all subjects (Fig. 3).

**Fig. 4:**
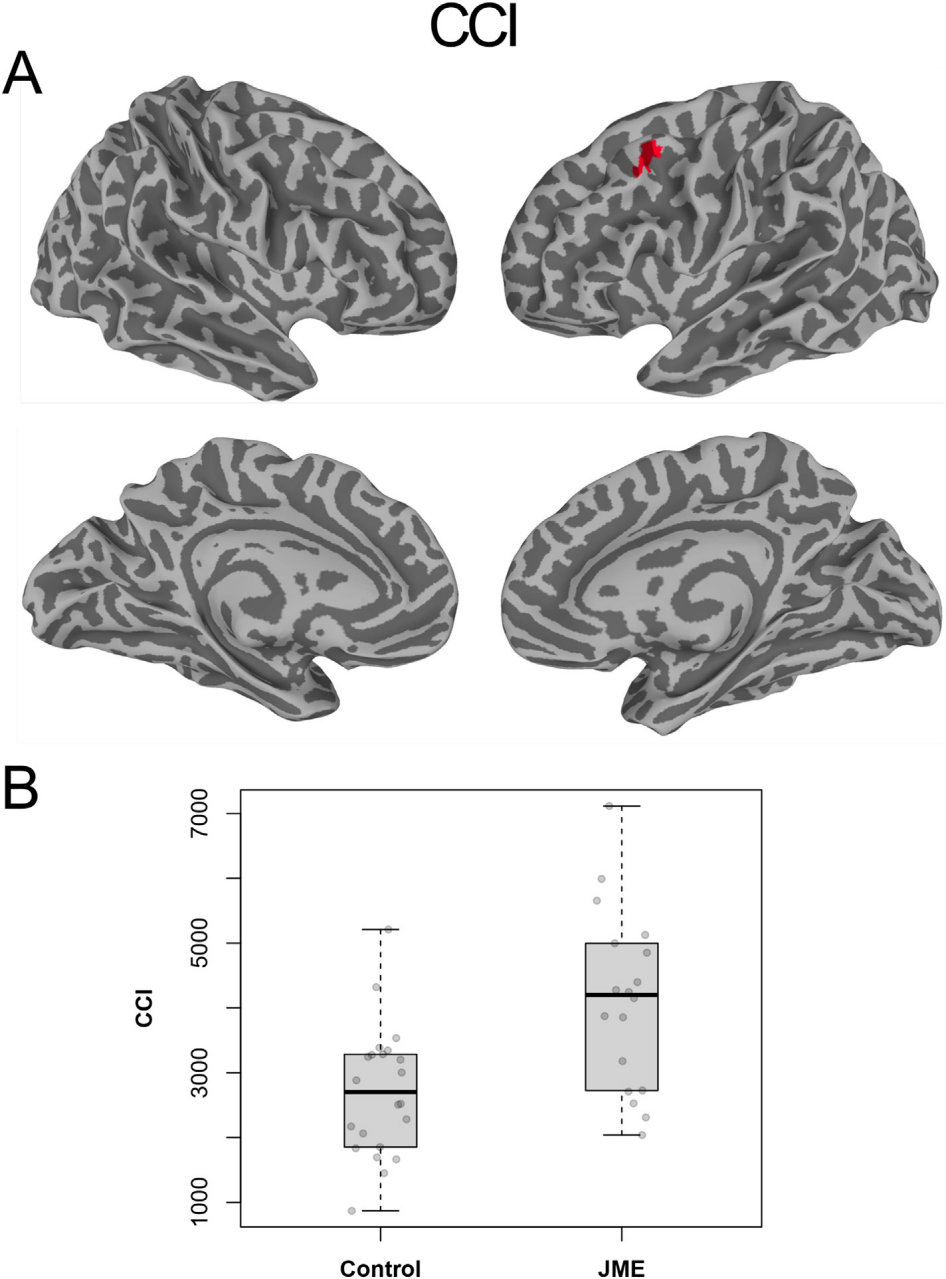
(A) Brain regions with statistical significant difference (Global FPR <5%) in CCI between healthy controls and JME patients; (B) Boxplots of the average CCI values for the individual subjects in the brain regions of interests depicted in (A). Cluster color encoding is arbitrary after ETAC thresholding.

**Fig. 5:**
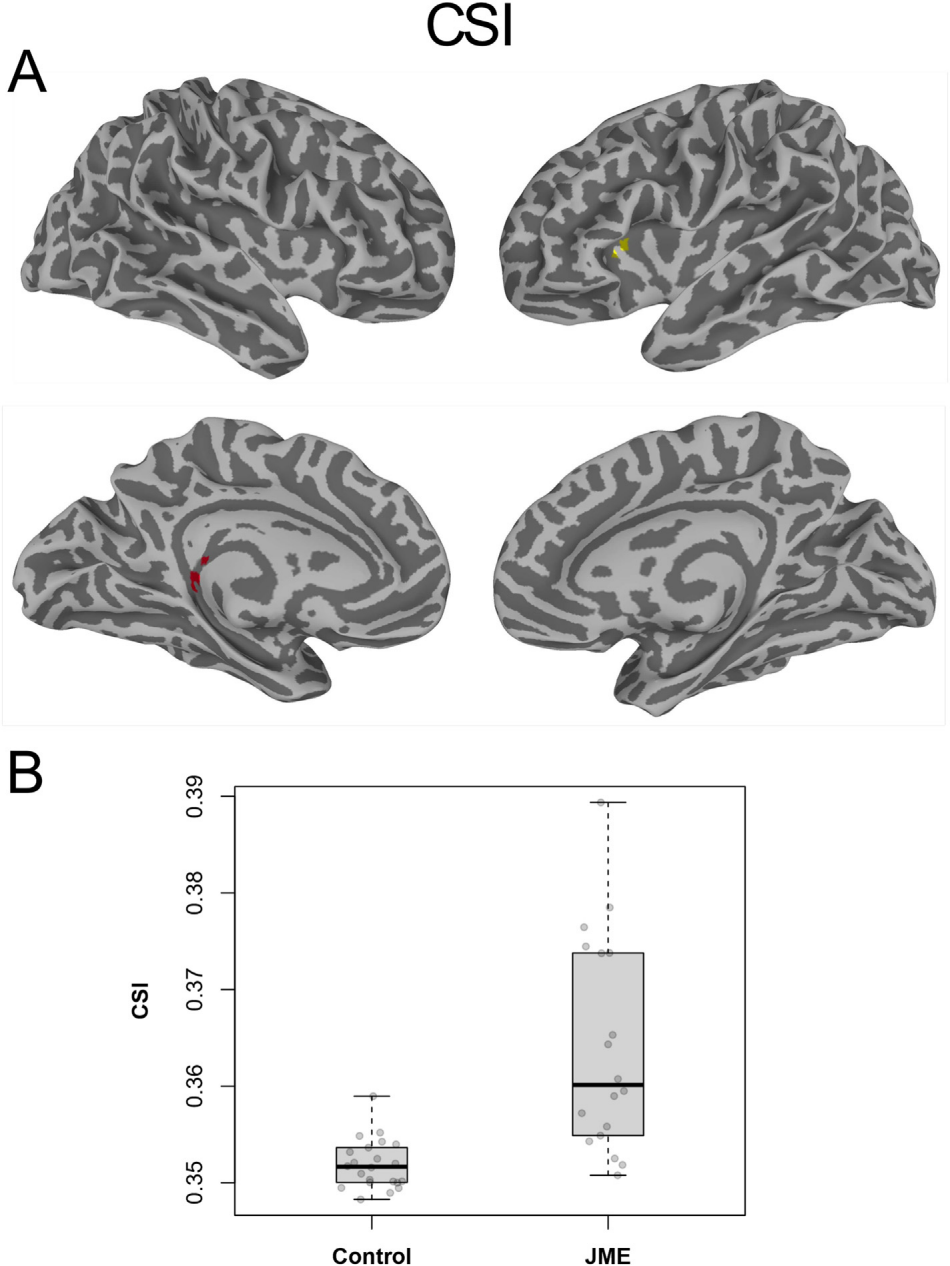
(A) Brain regions with statistical significant difference (Global FPR < 5%) in CSI between healthy controls and JME patients; (A) Boxplots of the average CSI values for the individual subjects in the brain regions of interests depicted in (A) with significant group difference. Cluster color encoding is arbitrary due to ETAC enhancement.

Fig. 4, Fig. 5 depict the results from the 2-way, unpaired Welch's *t*-tests of the static functional connectivity metrics, CCI and CSI based on the entire 10-min time courses, respectively. As detailed in Table 1, both CCI and CSI are significantly (Global FPR < 5%) higher for the JME patients in multiple brain regions. The involved brain areas are mainly in the left hemisphere, including the dorsolateral prefrontal cortex, dorsal striatum, precentral and middle temporal gyri. Compared with the normal controls, the ROI averages of the CSI and CCI metrics for the JME patients were about 3.2 and 52% higher, respectively (see Table 2, Figs. 4B and 5B).

**Table 1:** Summary of brain regions with significant group differences (Global FPR < 5%) in static metrics, CCI and CSI, as well as their corresponding dynamic metrics: <CCI> and <CSI>, between healthy controls and JME patients. The overlaps among any of these connectivity metrics are also detailed.

	Cluster Size	Center of Mass	Anatomical Location (Brodmann Area)
CCI	20	-41L, 16A, 45S	L. Middle Frontal Gyrus (BA8,9)
CSI	41	-21L, 11A, 2S	L. Lentiform Nucleus, L. Putamen
	38	-18L, -28P, 18S	L. Caudate, L. Thalamus
<CCI>	21	-41L, 17A, 43S	L. Middle Frontal Gyrus (BA8,9)
<CSI>	34	31R, 48A, -12I	R. Middle Frontal Gyrus (R. BA11)
	24	-47L, 27A, 2S	L. Inferior Frontal Gyrus (L. BA45)
	13	37R, -3P, -38I	R. Middle Temporal Gyrus (BA20)
Overlaps			
CCI/<CCI>	16	-41L, 16A, 44S	L. Middle Frontal Gyrus (BA8,9)

**Table 2:** Summary of the group averages for the static and dynamic functional connectivity metrics in the brain regions (see Fig. 4, Fig. 5, Fig. 6, Fig. 7) with significant group differences (Global FPR < 5%) between healthy controls and JME patients. Also shown are group difference *p*-values between controls and JME for the metrics averaged across the brain regions ROIs.

Metrics	Controls	JME	Difference	Controls VS JME
	Mean ± Std	Mean ± Std	%	P-value
CCI	2700 ± 1000	4100 ± 1400	52	1e−3
CSI	0.35 ± 0.0038	0.37 ± 0.011	3.2	6e−4
<CCI>	7700 ± 800	8800 ± 950	14	8e−4
<CSI>	0.42 ± 0.0068	0.43 ± 0.012	3.3	1e−4
Overlap CCI/<CCI>				
CCI	2700 ± 980	4100 ± 1400	53	9e−4
<CCI>	7700 ± 800	8800 ± 960	14	7e−4

As demonstrated in Fig. 4, Fig. 5, CSI is a more sensitive functional metric reflecting the hyper functional synchrony in the JME patients. The brain region with significant CSI difference was about 4 times of that for CCI (see Table 1, 79 voxels versus 20 voxels). There were no overlapping voxels between the CCI and CSI results.

**Fig. 6:**
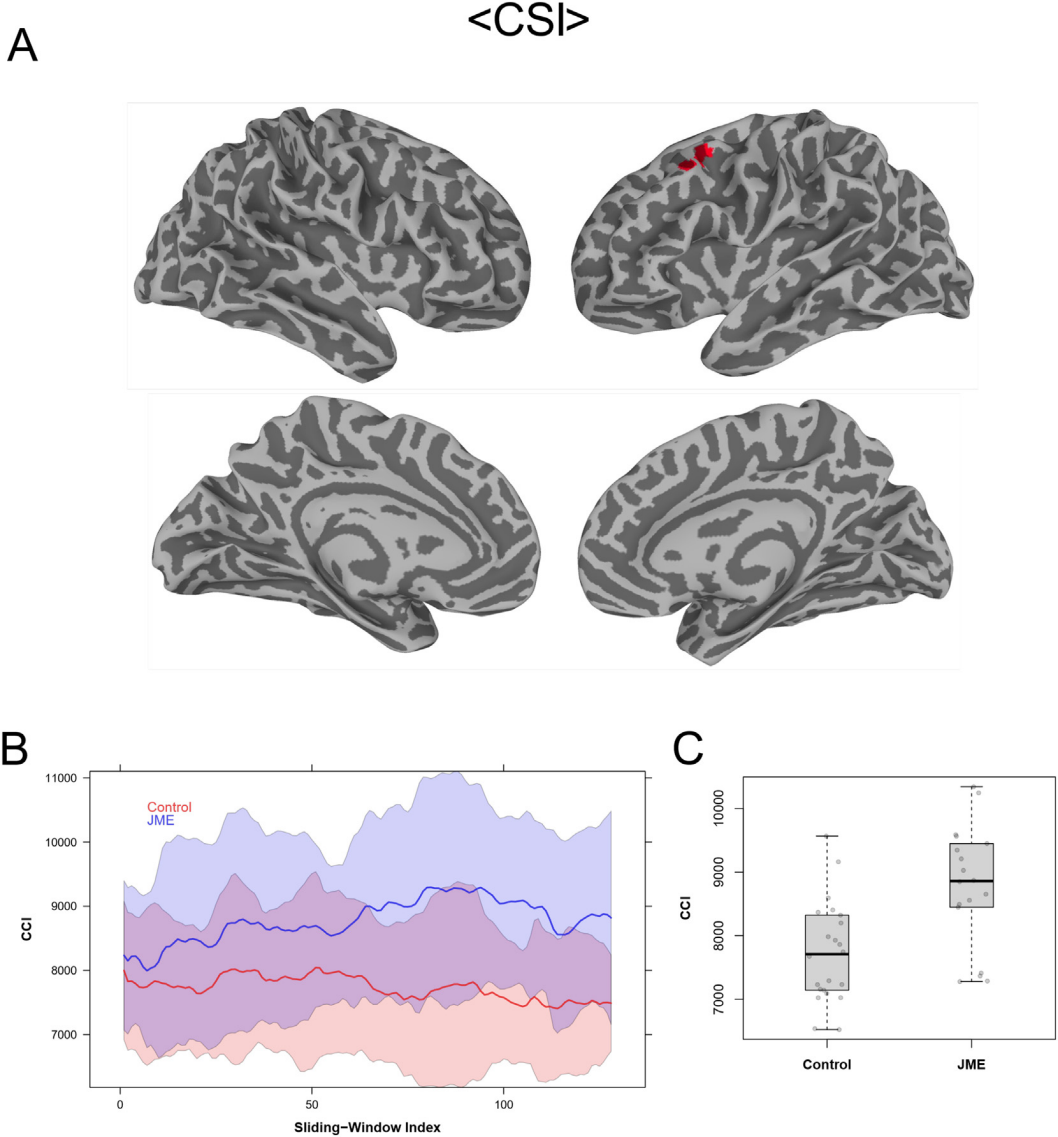
(A) Brain regions with statistically significant difference (Global FPR < 5%) in <CCI> between healthy controls and JME patients; (B) Mean CCI of each sliding window during the entire acquisition time period of JME patients and healthy controls respectively. The shaded regions correspond to the standard deviation of respective group; (C) Boxplots of the average < CCI> values for the individual subjects in the brain regions of interests depicted in (A) with significant group difference.

**Fig. 7:**
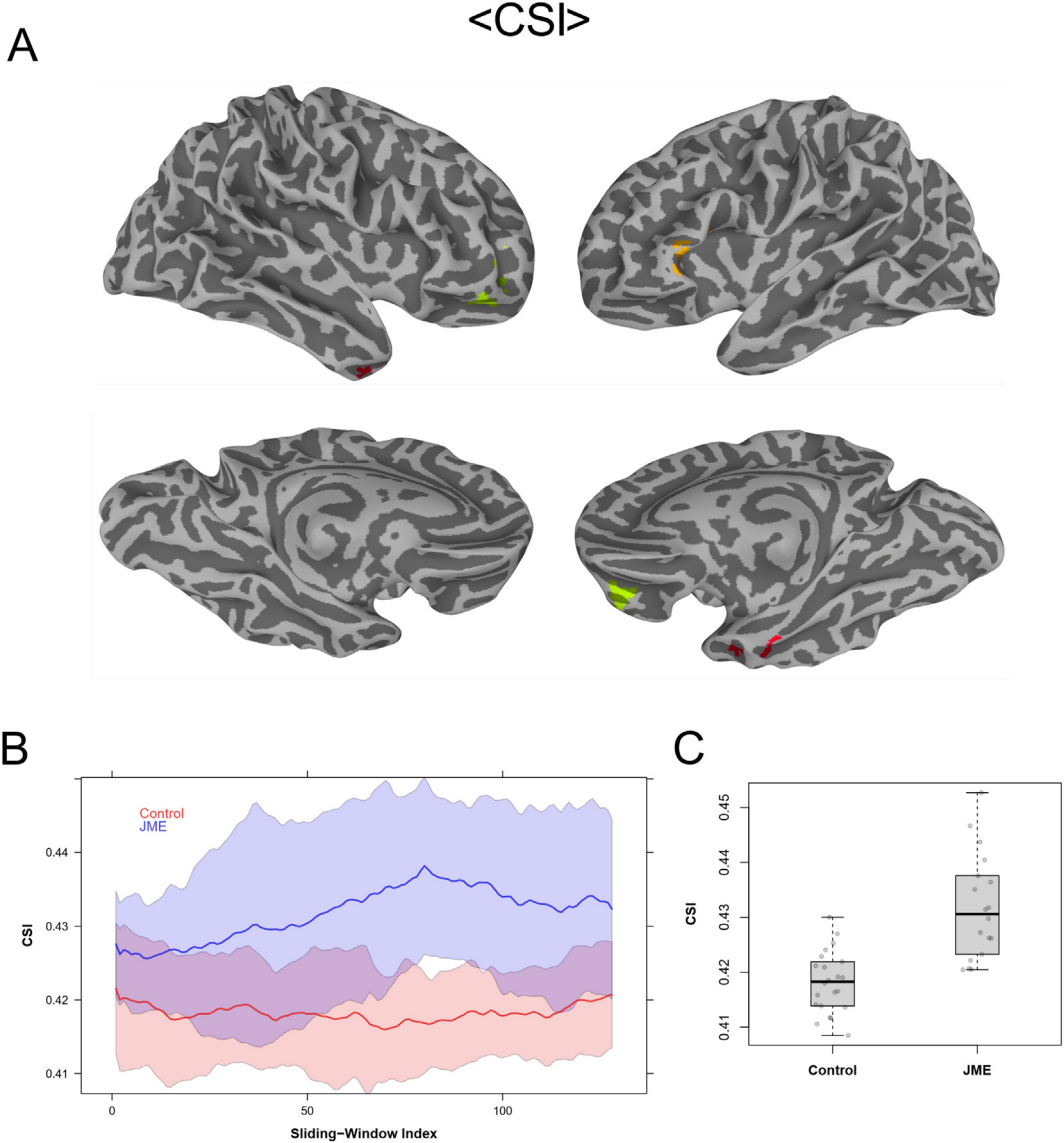
(A) Brain regions with statistical significant difference (Global FPR < 5%) in <CSI> between healthy controls and JME patients; (B) Mean CSI of each sliding window during the entire acquisition time period of JME patients and healthy controls respectively. The shaded regions correspond to the standard deviation of respective group; (C) Boxplots of the average <CSI> values for the individual subjects in the brain regions of interests depicted in (A) with significant group difference.

The <CCI> and <CSI> data from the 2-min sliding window revealed also multiple brain regions with significant (Global FPR < 5%) differences in dynamic functional connectivity metrics between the JME patients and healthy controls. Fig. 6, Fig. 7 depict the *t*-test results of the <CCI> and <CSI> data, respectively. Both <CCI> and <CSI> are significantly (Global FPR < 5%) higher in the JME patients in multiple brain regions. As detailed in Table 1, the involved brain regions are also primarily in the left hemisphere, including the dorsolateral prefrontal cortex, middle temporal gyrus and dorsal striatum.

Figs. 6B and 7B show the average time courses of the sliding window time series for CCI and CSI, respectively, corresponding to the ROIs where <CCI> and <CSI> are significantly different (Global FPR < 5%) between JME patients and normal controls. Comparing to the results for healthy controls, the time courses showed that JME patients have not only higher average values over the entire time period (i.e. higher <CCI> and <CSI>), but also higher level of variations as indicated by the standard deviations of the time series. The ROI averages of <CSI> and <CCI> for the JME patients were about 3.3 and 14% higher than those for the normal controls (see Table 1, Figs. 6C and 7C, respectively).

**Fig. 8:**
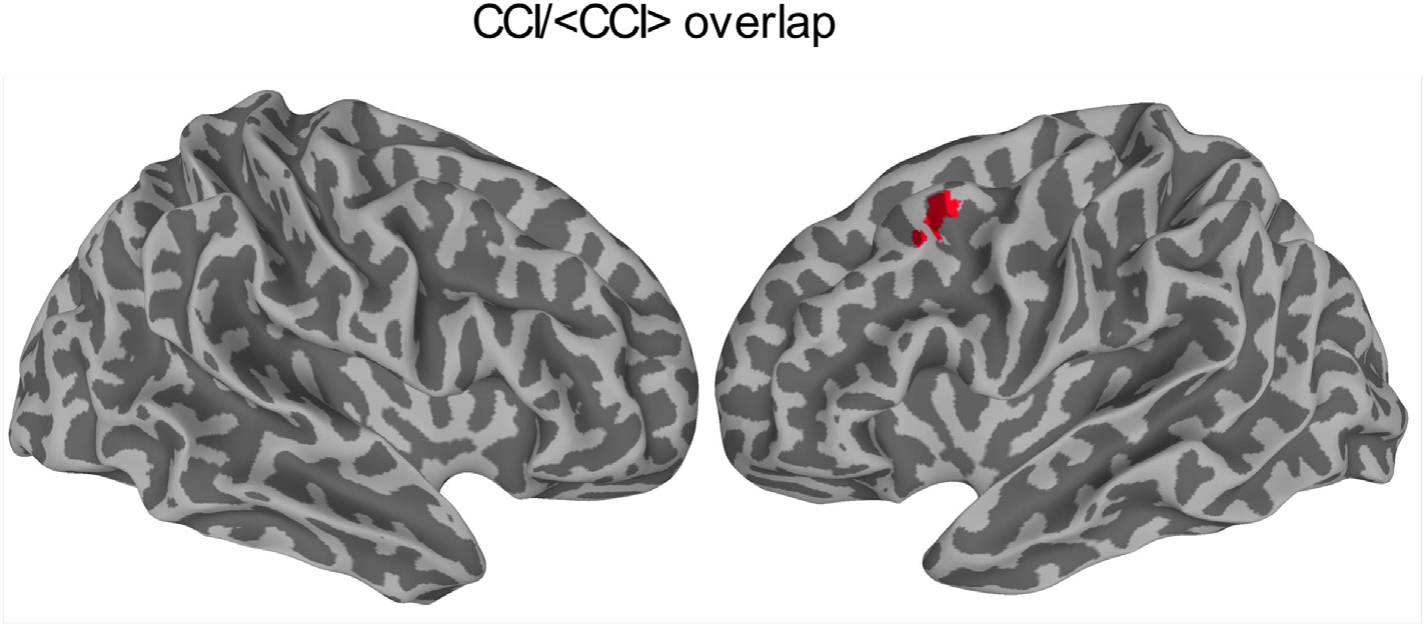
Overlapping brain regions between static CCI results shown in Fig. 4 and dynamic <CCI> results shown in Fig. 6.

Fig. 8 depicts the overlapping brain areas between the static and dynamic functional connectivity results. As detailed in Table 1, there were only 16 overlapping voxels between the CCI and <CCI> results, whereas there none for CSI and <CSI>. As detailed in Table 2, in this overlapping brain region, all the static and dynamic functional connectivity metrics were systematically higher for JME patients compared with healthy controls.

## 4 Discussion

In this study, we incorporated a 2-min sliding-window into the previously developed quantitative data-driven analysis framework to investigate the static and dynamic functional connectivity abnormalities in JME patients. The key findings of the study are the followings:

1) The static metrics, CCI and CSI, based on quantitative data analysis of the entire 10-min acquisition window of the Resting-state functional MRI data revealed significant higher static functional connectivity in JME patients compared with healthy controls in dorsal striatum and middle frontal gyri.
2) The dynamic metrics, <CCI> and <CSI> derived from 2-min SWATCH analysis, demonstrated also significant hyper functional connectivity dynamics dominant in the left hemisphere in multiple brain regions including dorsolateral prefrontal cortex, middle temporal gyrus and dorsal striatum.
3) The brain regions with abnormal functional connectivity density (CCI and <CCI>) are much smaller (one-half to a quarter) than those with hyper functional connection strength (CSI and <CSI>). Large portions of the abnormal functional connectivity density overlaps between the static and dynamic metrics, while the functional connectivity strength regions that show hyper-connectivity in JME patients do not overlap at all.

### 4.1 The quantitative data-driven analysis and SWATCH analysis methods

The quantitative data-driven analysis method has the following characteristics: It does not require prior parcellation of the Resting-state functional MRI data into anatomical regions, either using anatomical atlases or spatial independent component analysis (ICA). It is data-driven and hence does not require user input. For each dataset, quantitative data-driven analysis generates functional connectivity metrics based on voxel-wise Person's correlation coefficients, which can facilitate direct statistical comparison between subject groups. To capture the abnormalities in dynamic functional connectivity for the JME patients, we extended our existing framework to incorporate a 2-min sliding window and evaluated the temporal average of the time-varying functional connectivity metrics.

Different approaches have been used to study the temporal variations of the resting-state brain functional connectivity, ranging from sorting of resting-state functional MRI data into distinct co-activation patterns (Liu and Duyn, 2013) to more sophisticated temporal ICA on top of spatial ICA (Smith et al., 2012). Time-frequency decomposition using the wavelet transform (Torrence and Compo, 1998) has also been successfully applied to investigate the temporal variations of the RFNs (Yaesoubi et al., 2015). The sliding-window approach is intuitive and commonly used (Allen et al., 2014; Chang and Glover, 2010; Handwerker et al., 2012; Jones et al., 2012; Kiviniemi et al., 2011; Hutchison et al., 2013b). It can be readily incorporated with well-established analysis methods for resting-state functional MRI data, such as, ICA (Allen et al., 2014; Chang and Glover, 2010; Sakoglu et al., 2010) and ROI seed methods (Chang and Glover, 2010; Handwerker et al., 2012).

The sliding-window approach was also opted here to assess the dynamic characteristics of RFNs, as it is straightforward to be combined with our previously developed quantitative data-driven analysis framework (Li et al., 2016; Nordin et al., 2016). However, there are a number of known issues with sliding-window approaches (Hutchison et al., 2013a; Hindriks et al., 2016), particularly regarding to the window size. Empirical evidence from previous studies suggests that 1-min window size is sufficiently robust to detect functional connectivity networks corresponding to distinct cognitive states (Shirer et al., 2012) and brain network topology (Jones et al., 2012) in typical fMRI scans. The loss of signal-to-noise-ratio (SNR) becomes an issue of concern for shorter window sizes, as there are fewer time points available in each window and the higher frequencies will be increasingly dominate. Overall this will increase the variability in the sliding-window metrics independent of neuronal activity. If the sliding window and sliding step are large, the temporal resolution will be too low. After a systematic testing with different window sizes (detailed in Supplementary Information B), we choose to use a 2-min window size and the minimum sliding increment of 1 timeframe for the present study to achieve sufficient SNR for the dynamic functional connectivity metrics without losing temporal resolution.

Static metrics provide an ‘average’ picture of brain network dynamics. By quantifying dynamic metrics, we obtain a view of the brain's dynamic interactions (Hutchison et al., 2013). This is especially useful when studying JME subjects with paroxysmal characteristics. We used the temporal averages, <CCI> and <CSI>, to assess the dynamic characteristics of their corresponding sliding-window time series for CCI and CSI. However, they are still rudimentary measures and do not fully capture the nature of temporal dynamics. We conducted extensive testing of different measures that can assess the temporal dynamics of a sliding window time series, including standard deviation, variance, and entropy. The temporal averages were opted as the measure of choice in this study because they are robust with sufficient SNR and sensitive to detect the dynamic functional connectivity differences between JME patients and normal volunteers.

Larger clusters formed through dynamic compared to static functional connectivity may be attributed to the overall larger values in the dynamic metrics as compared to the static, but more likely due to slight differences in the distribution of values, where there is a larger portion of large values for dynamic metrics as compared to the static as seen in Fig. 3. However, there is no overlap between the static and dynamic connectivity strength indices, suggesting that while our dynamic approach may reveal additional information, it cannot be solely to replace the static. Multi-length sliding-window approaches are currently being investigated to capture the slower variations in dynamic functional connectivity.

### 4.2 Neuropathological characteristics of JME

Since its initial identification in 1957, JME has now been widely recognized as a common epilepsy form with brain function network abnormality via a variety of underlying causes (Yacubian, 2017; Santos et al., 2017). Typical clinical characteristics of JME include the following: 1) Myoclonic jerks of the upper limbs, tonic-clonic seizures and infrequent absence seizures; 2) a lack of apparent structural pathology in MRI and computer tomography; 3) close association with cognitive and emotion deficiency indicative of frontal lobe dysfunctions. Identifying the precise JME specific abnormalities in brain functional networks has been considered as the key for understanding neural mechanisms underlying the cognitive impairments. Neuroimaging studies in last two decades have provided several lines of evidence suggesting that JME is characterized by common brain functional network abnormalities despite the heterogeneous nature of this patient group. For example, positron emission tomography (PET) demonstrated metabolic and neurotransmitter changes in the dorsolateral prefrontal cortex and midbrain structures reflecting the particular cognitive and behavioral profile of JME patients (Anderson and Hamandi, 2011; Meschaks et al., 2005; Ciumas et al., 2008). 1H magnetic resonance spectroscopy (1H -MRS) has shown evidence of progressive frontal and thalamic dysfunction (Zhang et al., 2016; Savic et al., 2004). BOLD fMRI studies have also shown that JME patients have aberrant functional integration between the motor and frontal networks (Vollmar et al., 2011). More recent resting-state functional MRI studies have further shown that JME patients depict hyper synchronized RFNs in multiple brain regions involving mainly the thalamo-prefrontocortical networks (Dong et al., 2016b; Jiang et al., 2018).

### 4.3 Hyper dynamic functional connectivity in JME

To the best of our knowledge, this is the first study to demonstrate abnormalities in dynamics of RFNs in a group of JME patients. Several studies have previously shown similar behavior for TLE (Laufs et al., 2014; Nedic et al., 2015; Douw et al., 2015; Robinson et al., 2017). Taken together with the literature results from static functional connectivity studies, our finding provides further evidence for ‘common’ fronto-cortical-subcortical network abnormalities among subjects with clinically and genetically heterogeneous JME. This is also supported by the results from neuropsychological studies (Yacubian, 2017; Santos et al., 2017), which revealed cognitive deficits in patients with JME, mainly implicating the thalamo-frontocortical network dysfunction.

## 5 Limitations of the study

It remains important to test the overall specificity of the brain network changes we have observed in this cohort of JME subjects. Recent dynamic functional connectivity studies of TLE patients have reported the engagement of similar but not identical networks (Laufs et al., 2014; Nedic et al., 2015; Douw et al., 2015). However, this appears not valid for the generalized epilepsies. A limitation of this study is that we were not able to account for functional brain changes potentially caused by anti-epileptic drug use (Fingelkurts et al., 2004; Wang et al., 2012; Fraschini et al., 2014; Hermans et al., 2015; Delahaye-Duriez et al., 2016). However, it is unlikely that our results can be accounted for by anti-epileptic drug effect at group level considering the different types of drugs and years of treatment (see Supporting Information A).

Since the current sample of JME patients did not perform MRI scans during seizure, it is unlikely that our findings indicate network properties that are directly involved in instigation or propagation of seizures.

To better characterize the brain regions displaying abnormal functional connectivity between JME patients and healthy controls. Whether the hyperactive brain regions have a specific target in their hyperactive states in the form of specific functional connectivity network, or another brain region, is not characterized in this study. Neither EEG, nor PET/SPECT data were acquired as a part of this study. Hence it is difficult to draw any definitive conclusions concerning the exact nature of any abnormalities detected in static, or dynamic, functional connectivity from the resting-state fMRI data presented here since we cannot confirm any potential hypothesis for their underlying mechanisms. Also, without complementary imaging modalities, we cannot definitively rule out the possibility that the brain regions shown to be abnormal in functional connectivity in our analysis are not false positives. We have attempted to confirm the validity of each resulting cluster with published literature and have found that all brain regions found to be abnormal between JME patients and healthy controls in this study support existing literature on the disease. While this does not constitute as definitive proof that our results are not, at least in part, contaminated by false positives, it does give indication that our analysis methods show promise in detecting true functional connectivity abnormalities.

The presented clusters in in this study undergone filtering through both cluster simulation and ETAC thresholding, which is somewhat redundant and overly zealous to guarantee global false positive rates <5%, we are currently looking into alternative analysis methods in conjunction with cluster enhancement methods (Smith and Nichols, 2009) to control global false positive rates in a more consistent fashion. This is especially impactful when the results as presented here consists mostly of small focal areas, of which some are sub-cortical. Our acquisition method is optimized for detecting abnormalities in large cortical regions foremostly, and due to the spatial resolution, not suited for spatially defining focal regions. By strict control of global false positive rates in the statistical analysis, we sacrifice spatial definition for robustness. Further studies, hopefully in conjunction with other modalities such as EEG and PET/SPECT, should be undertaken with higher spatial resolution to more clearly define structure, and substructure, boundaries.

## 6 Conclusion

In summary, neuroimaging studies have provided multiple lines of evidence to support the notion that JME is a frontal lobe variant of a multi-regional, thalamocortical ‘network’ epilepsy form, rather than a generalized epilepsy syndrome. Incorporating a 2-min sliding window approach into quantitative data-driven analysis of resting-state functional MRI data can reveal additional information on the temporally fluctuating functional connectivity networks of the human brain, which indicate that RFNs involving dorsolateral prefrontal cortex have unique hyper dynamic characteristics in JME patients. Accessing the static along with the dynamic functional connectivity analysis may provide additional information for temporally transients diseases such as epilepsy, providing further insights into the abnormalities in functional connectivity underlying the cognitive deficiency in JME.

## Appendix A Supplementary data

Patient information Image 1

Determining sliding window length. Image 2
