# Supplementary material for: Juvenile myoclonic epilepsy has hyper dynamic functional connectivity in the dorsolateral frontal cortex
Source: Neuroimage Clin. 2018 Nov 19;21:101604. doi: 10.1016/j.nicl.2018.11.014 (PMC6412974; doi:10.1016/j.nicl.2018.11.014)
Supplement: Supplementary file 1 — Patient information [file mmc1.docx]

# Supporting Information A: Summary of the demographic information of the participating patients.

Table A: Patient information. Total seizures refer to the total number of documented seizures prior to MRI scanning. Medication is the actively prescribed medication at the time of MRI scanning session.

| Patient ID | Gender | Handedness | Heredity | Age | Onset Age | Total seizures | Seizure frequency (per year) | Medication |
| --- | --- | --- | --- | --- | --- | --- | --- | --- |
| JME01 | Male | Right | Probably | 39 | 12 | <10 | <1 | Valproate |
| JME02 | Female | Right | No | 22 | 13 | 6 | <1 | Lamotrigin |
| JME03 | Female | Right | No | 30 | 12 | 20 | Varies | Carbamazepine |
| JME04 | Female | Right | No | 35 | 13 | 6 | <1 | Valproate, Levetirazetam |
| JME05 | Female | Right | No | 20 | 10 | 3 | <1 | Valproate |
| JME06 | Male | Right | Yes | 23 | 15 | 10 | <1 | Valproate |
| JME07 | Female | Right | No | 48 | 14 | 20 | <1 | Valproate, Levetirazetam |
| JME08 | Female | Ambi | Yes | 30 | 11 | 10 | <1 | Valproate |
| JME09 | Male | Right | Unknown | 38 | 12 | 10 | <1 | Valproate |
| JME10 | Female | Right | No | 21 | 13 | 6 | <1 | Levetriazepam |
| JME11 | Female | Right | No | 30 | 12 | 50 | 5 | Valproate |
| JME12 | Female | Right | Yes | 32 | 10 | 50 | 3 | Lamotrigin, Levetirazepam |
| JME13 | Female | Right | No | 30 | 19 | 120 | 12 | Levetriazepam |
| JME14 | Female | Right | Yes | 23 | 11 | 20 | <1 | Lamotrigin, Levetriazepam |
| JME15 | Female | Right | No | 41 | 13 | 50 | 3 | Lamotrigin, Valproate |
| JME16 | Female | Right | Yes | 28 | 16 | 15 | <2 | Orfiril long |
| JME17 | Female | Right | No | 23 | 17 | 4 | <1 | Valproate |
| JME18 | Female | Right | Yes | 29 | 16 | 2 | <1 | Absenor |
