# Supplementary material for: Juvenile myoclonic epilepsy has hyper dynamic functional connectivity in the dorsolateral frontal cortex
Source: Neuroimage Clin. 2018 Nov 19;21:101604. doi: 10.1016/j.nicl.2018.11.014 (PMC6412974; doi:10.1016/j.nicl.2018.11.014)
Supplement: Supplementary file 2 — Determining sliding window length. [file mmc2.docx]

# Supporting Information B: Determining window length for the sliding-window analysis

## Estimation

Decreasing the window-length will naturally decrease the amount of signal in each window, while increasing the window length decrease the amount of temporal dynamic information available. Since our purpose is to incorporate temporal dynamic information in our analysis, we wish to have shortest possible window length to capture dynamic information throughout our acquisition, but long enough to provide consistently high enough SNR to calculate our metrics reliably.

To determine the shortest window length possibly while preserving some signal to compute our metrics, we computed the SNR, as defined by $SNR=\frac{\mu}{\sigma}$), for sliding-window lengths ranging from 30 seconds to 6 minutes in 10 randomly selected subjects (5 patients and 5 controls). The average SNR for all sliding-windows from the beginning of the acquisition to the end was chosen as an estimate of the sliding-window SNR (${SNR}_{SW}$) present in each voxel. To minimize computation time, we opted to include only voxels in a gray-matter mask.

The average sliding-window SNR over all voxels in the gray-matter mask can be seen in Figure s1A. Consistent with published literature on the matter, window lengths below approximately 1 minute will very quickly drop in SNR, most likely because there simply isn’t enough signal in the window. However, the SNR steadily increases above the 1-minute window length. This is not surprising either since by adding more data per window, we increase the SNR on average. Not only do we strive for sufficiently high SNR on average, we also desire consistently high SNR throughout the brain (gray-matter) to reliably calculate our metrics. Figure s1B shows the standard deviation of the sliding-window SNR over all voxels in the gray-matter mask. As expected, the standard deviation does decrease with increasing window length. The standard deviation decreases greatly with increase in window length for short window length, but the gains in homogeneity is diminished after a certain point. From this we decided our window length to be 2-minutes, after which we do no longer gain proportionally in homogeneity in sliding-window SNR over the gray-matter.


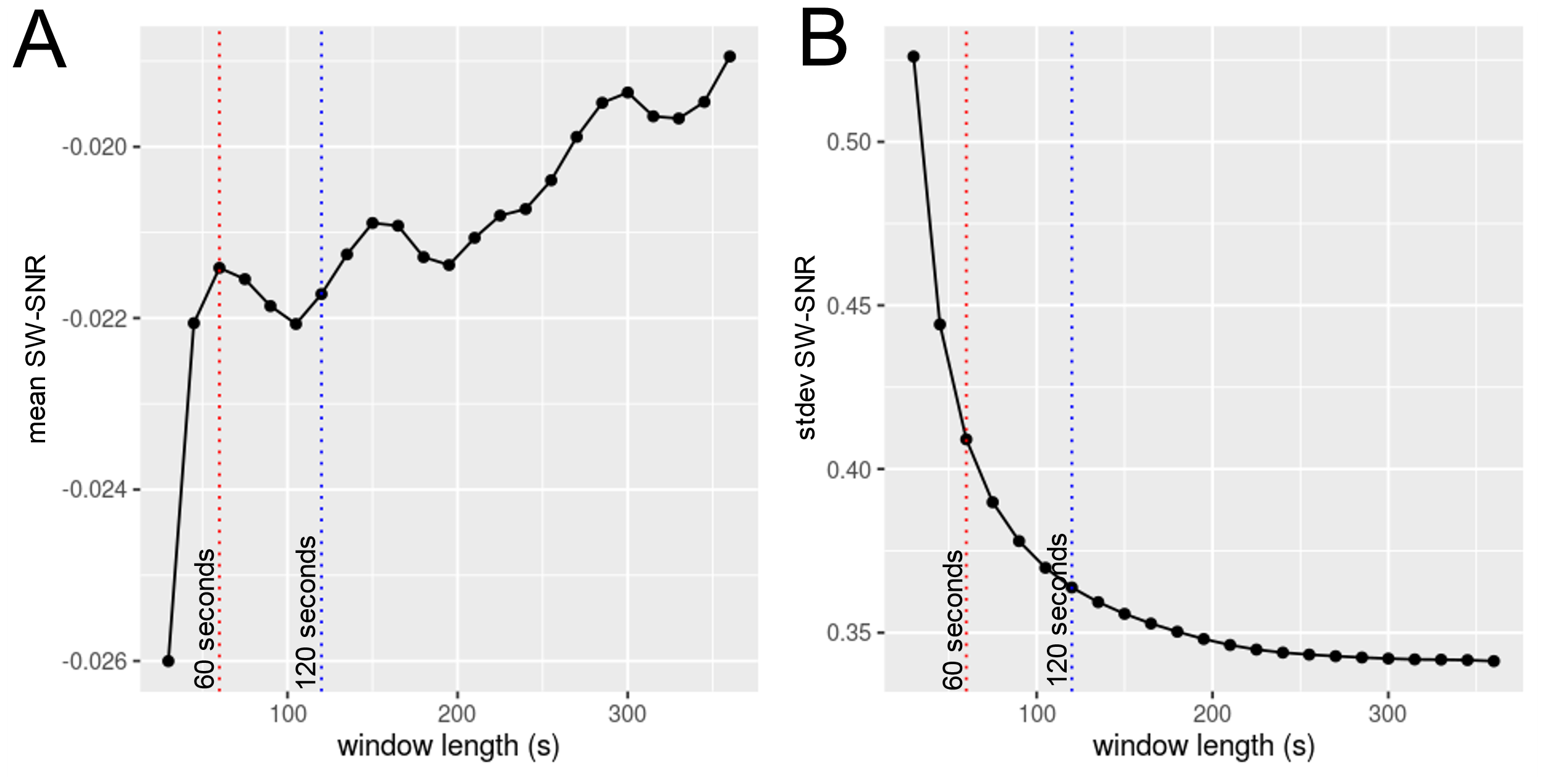


***Figure s1: A: mean sliding-window SNR of all voxels inside gray-matter mask over a range of window lengths. B: Standard-deviation of sliding-window SNR of all voxels inside gray-matter mask over a range of window lengths. The mean SNR deteriorates quickly below a window length of 60 seconds and increases steadily above that. The standard deviation decreases quickly from very short window lengths, but the rate drops off quickly, meaning there is relatively little to be gained in terms of obtaining homogenously high SNR throughout gray-matter with excessive increases in window-length. A 2-minute window was chosen to obtain high SNR while, but short enough such as any further increase in window-length doesn’t result in high gains in homogeneity of SNR throughout gray-matter.***

## Confirmation

To confirm that our selection of window length does result in robust metrics that provides decent time varying dynamic information, we systematically calculated our dynamic metrics using varying window lengths in the same range as before.

To access our resulting metrics in terms of dynamic information content, we calculated the coefficient of variance (CV) according to $CV=\frac{1}{SNR}= \frac{\sigma}{\mu}$ to access the temporal variance. Figures s2A and s2D shows the coefficient of variance of CCI and CSI respectively at different window lengths. The results for dynamic CSI showed consistent decrease in coefficient of variance throughout our range. This is expected as increasing our window length increases the amount of temporal smoothing on the dynamic metrics, and hence decreasing temporal variations. What is more interesting to note is that the dynamic CCI increases in its coefficient of variance from 30 seconds up until approximately 2 minutes before decreasing again. This initial low coefficient of variance, and its increase until the 2-minute window length before decreasing again may be due to the low SNR of the resting-state fMRI data at these low window lengths. Figure s2A shows that our choice of a 2-minute sliding window length for this study is suitable as we succeeded preserving temporal variation in our metrics, while avoiding excessive loss in SNR.


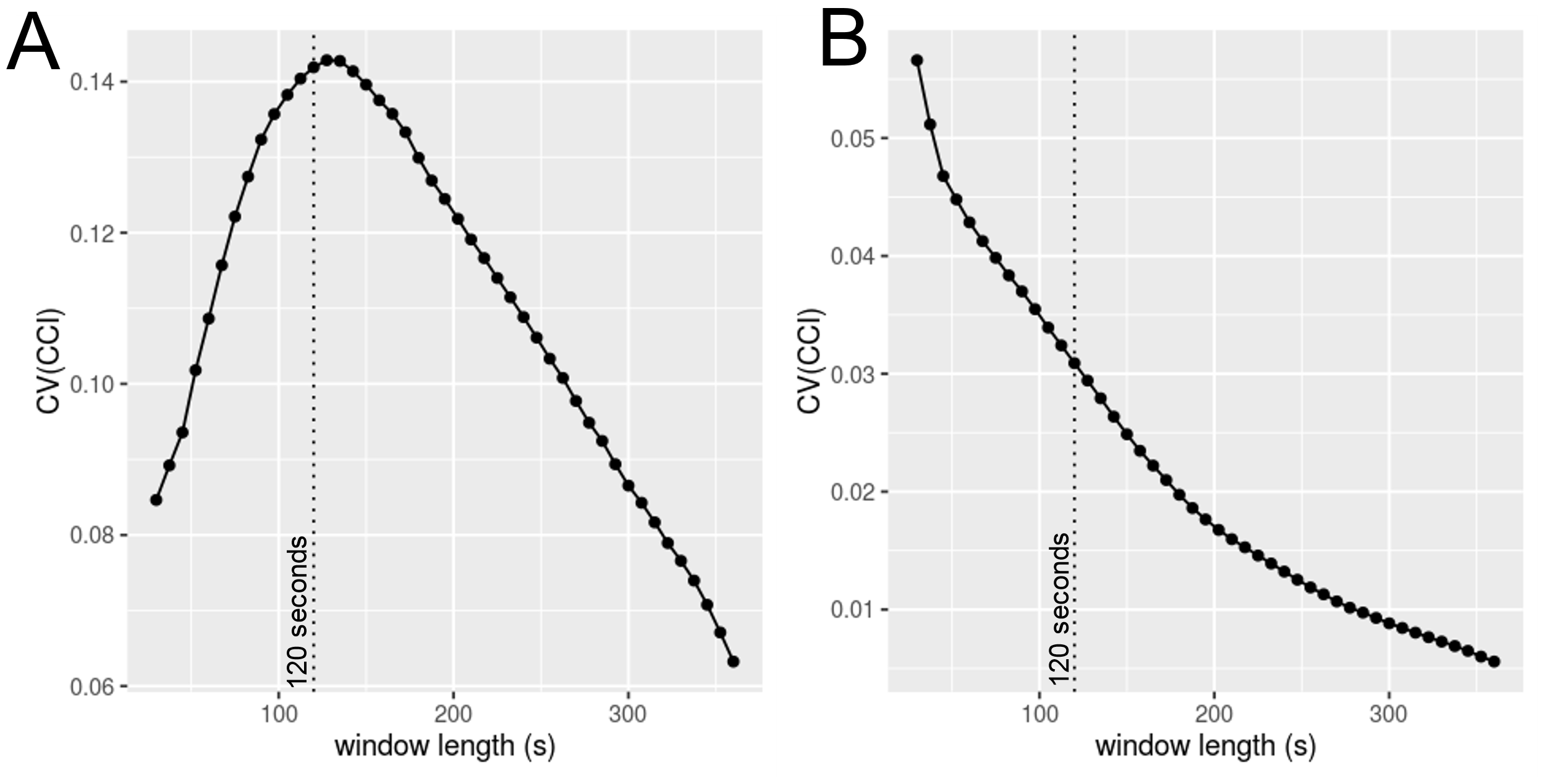


***Figure s2: A: Coefficient of Variance of CCI at varying window lengths. B: Coefficient of Variance of CSI at varying window lengths. As expected, longer window lengths result in less variance in the metric time-courses. An exception is at low window lengths, where CCI unexpectedly increase in variance, maximizing at approximately 2-minutes in window length before decreasing.***
